# Economic and clinical burden associated with respiratory viral infections after allogeneic hematopoietic cell transplant in the United States

**DOI:** 10.1111/tid.13866

**Published:** 2022-06-01

**Authors:** Michael G. Ison, Francisco M. Marty, Nelson Chao, Seung Hyun Moon, Zhiji Zhang, Aastha Chandak

**Affiliations:** ^1^ Northwestern University Feinberg School of Medicine Chicago Illinois USA; ^2^ Brigham and Women's Hospital Boston Massachusetts USA; ^3^ Duke University Medical Center Durham North Carolina USA; ^4^ AlloVir Cambridge Massachusetts USA; ^5^ Certara New York New York USA

**Keywords:** allogeneic HCT, clinical burden, health care burden, respiratory viral infection

## Abstract

**Background:**

Allogeneic hematopoietic cell transplant (allo‐HCT) recipients are at increased risk for respiratory viral infections (RVIs), which invoke substantial morbidity and mortality. Limited effective antiviral options and drug resistance often hamper successful RVI treatment, creating additional burden for patients and the health care system.

**Methods:**

Using an open‐source health care claims database, we examined differences in clinical outcomes, health resource utilization, and total reimbursements during the 1‐year period following allo‐HCT in patients with and without any RVI infection (respiratory syncytial virus, influenza, parainfluenza virus, and human metapneumovirus). RVIs were diagnosed at any time ≤1 year after allo‐HCT and identified by International Classification of Disease codes. Analyses were stratified by the presence or absence of acute or chronic graft‐versus‐host disease (GVHD).

**Results:**

The study included 13 363 allo‐HCT patients, 1368 (10.2%) of whom had a diagnostic code for any RVI. A higher proportion of patients with any RVI had pneumonia ≤1 year after allo‐HCT compared to patients without any RVI, with or without GVHD. Patients with any RVI had higher all‐cause mortality risk, longer length of post‐allo‐HCT hospital stay, higher readmission rate, and higher number of hospital days after allo‐HCT compared to patients without the infection (all *p *< .05). Total unadjusted median reimbursements were higher for those with any RVI and each specific RVI assessed than those without the specific infection, with or without GVHD.

**Conclusion:**

Allo‐HCT patients with RVIs had significantly worse clinical outcomes and increased health resource utilization and reimbursements during the year following allo‐HCT, with or without GVHD.

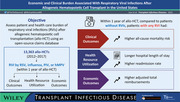

**Abbreviations**
allo‐HCT: allogeneic hematopoietic cell transplant
DRG: Decision Resource Group
GVHD: graft‐versus‐host disease
hMPV: human metapneumovirus
HR: hazard ratio
ICD: International Classification of Diseases
LOS: length of stay
LRTI: lower respiratory tract infection
PIV: parainfluenza virus
RSV: respiratory syncytial virus
RVI: respiratory viral infection
URTI: upper respiratory tract infection

## 1 INTRODUCTION

Allogeneic hematopoietic cell transplant (allo‐HCT) recipients are at an increased risk for respiratory viral infections (RVIs),^1^ with respiratory syncytial virus (RSV) being the most common cause of RVI in this population (incidence of up to 50%), followed by influenza (up to 40%), parainfluenza virus (PIV; up to 27%), and human metapneumovirus (hMPV; up to 11%).^2^ RVIs are associated with substantial morbidity and mortality in HCT recipients, often related to the progression from an upper respiratory tract infection (URTI) to a lower RTI (LRTI).^2^, ^3^, ^4^, ^5^, ^6^, ^7^, ^8^, ^9^, ^10^ As with other types of infections, RVIs occur more frequently after allo‐HCT than after autologous HCT,^11^, ^12^ owing to delayed immune reconstitution^13^ and lack of humoral and T‐cell–mediated immunity.^1^, ^14^, ^15^ In addition to increased frequency, mortality associated with RVIs is higher after allo‐HCT than after autologous HCT.^16^ A limited number of effective antivirals and the emergence of drug resistance, likely due to prolonged viral shedding in the setting of antiviral monotherapy, often hamper successful treatment of RVIs.^1^, ^17^, ^18^ In order to better understand the clinical and economic impact of RVIs in allo‐HCT recipients, this retrospective observational study utilized real‐world claims database to compare clinical outcomes, health resource utilization, and health care reimbursement in allo‐HCT recipients with and without RVIs, including RSV, influenza, PIV, and hMPV.

## 2 METHODS

Patients who underwent allo‐HCT between January 1, 2012 and December 31, 2017 were identified from an open‐source claims database obtained from the Decision Resource Group (DRG) Real‐World Evidence Data Repository (now part of Clarivate). Data were available from January 1, 2011 to December 31, 2018 to ensure that all patients have at least 1‐year period prior to index procedure and 1‐year period after index procedure for follow‐up. The DRG Real‐World Evidence Data Repository is built upon an aggregation of pharmacy and medical billing clearinghouse claims that are updated daily from vendors. DRG Real‐World Evidence Data Repository covers numerous commercial and government health plans and tracks more than 300 million longitudinal patient lives. The open‐source nature of the database allows patients to be tracked as they change payers.

Patients were categorized according to the presence or absence of an RVI, defined as having ≥1 International Classification of Diseases (ICD‐9 or ICD‐10) diagnosis code for RSV, influenza, PIV, or hMPV at any time point for 1 year after allo‐HCT. First (index) allo‐HCT was identified through an ICD‐9 or ICD‐10 procedure code, a Current Procedural Terminology code, or a Healthcare Common Procedure Coding System code. For patients who had multiple allo‐HCTs during this period, only data from the first were included in the study to avoid double counting. Patients who underwent allo‐HCT in an outpatient setting were excluded in order to maintain a homogeneous cohort, as few patients undergo allo‐HCT in an outpatient setting and those that do tend to have different practice patterns. The baseline period was defined as the 1‐year prior to index allo‐HCT; patients were followed for 1 year after the procedure.

Clinical outcomes included presence of pneumonia and time to all‐cause mortality. Health resource utilization outcomes included overall hospital length of stay (LOS) for the index hospitalization and hospital readmissions, and readmission rate. Total health care reimbursement was also evaluated. Since adjudicated reimbursed claims information is not available for all submitted claims in an open‐source claims database, the available overlapping submitted and reimbursed claims—a 20% overlap in the study cohort—were used to calculate the reimbursement‐to‐charge ratio. This ratio was calculated as 0.425 and applied to the submitted charges to generate the reimbursement amount for the study cohort. These reimbursed amounts were then winsorized at the first and 99th percentiles to adjust for outliers in each group. Further, the total reimbursed amounts were identified in the 1‐year follow‐up period after allo‐HCT and were presented as 2019 US dollars (using the medical care component of the consumer price index).

### 2.1 Statistical analysis

For all study outcomes, patients were stratified by presence/absence of any RVI, as well as each type of RVI (RSV, influenza, PIV, and hMPV). Separate analyses were reported for those with acute or chronic graft‐versus‐host disease (GVHD) versus those without. Chi‐square test or Fisher exact test was applied to statistically compare pneumonia and all‐cause mortality rates. Time to all‐cause mortality was evaluated using an adjusted Cox proportional hazards model. Variables included baseline covariates of age at index hospitalization, underlying disease, stem cell source, and number of comorbidities; time‐varying covariates included RVI and GVHD based on their dates of diagnosis. Variables with *p *< .10 were retained in the final multivariate model.

We describe total health care reimbursement amounts, with adjusted reimbursement for patients with and without RVI estimated through a generalized linear model fitted with a negative binomial distribution. Adjustment covariates included age, health plan type, underlying disease, stem cell source, GVHD during follow‐up, interaction between presence of RVIs and GVHD, total reimbursements during the baseline period, and follow‐up time. As with time to all‐cause mortality, variables with *p *< .10 were retained in the final adjusted model. Observed‐margin least square means and 95% CIs were extracted as adjusted reimbursements stratified by the presence or absence of any GVHD after transplant. All statistical tests were two sided and at 5% level of significance. All analyses were conducted using SAS version 9.4 or later.

## 3 RESULTS

### 3.1 Study population

A total of 17 520 patients with first allo‐HCT procedure codes between January 1, 2012 and December 31, 2017, and no prior allo‐HCT procedures in the 1‐year prior, were identified within the database. Those without a corresponding allo‐HCT hospitalization (*n* = 2933) and who underwent index allo‐HCT in an outpatient setting (*n* = 1224) were excluded, leaving a total of 13 363 eligible for inclusion in the final study cohort (Figure S1). This final cohort represented approximately 22% of allo‐HCT recipients reported to the Center for International Blood and Marrow Transplant Research during that period. A total of 1368 (10%) patients were diagnosed with an RVI within the first year after allo‐HCT, including 687 (5%) with influenza, 578 (4%) with RSV, 181 (1%) with hMPV, and 166 (1%) with PIV. The average standard deviation time to first RVI infection after allo‐HCT was 161.2 (109.1) days; 226 patients were diagnosed with ≥2 RVIs during the year after transplantation. The average follow‐up time after allo‐HCT was 323.5 days for all patients, 337.4 days for patients diagnosed with an RVI, and 321.9 days for patients without an RVI diagnosis.

Demographic and transplantation characteristics of the patient population can be seen in Table S1. Mean ages of patients were 44 and 47 years in the group with and without infection, respectively; the majority were male and had malignant disease, peripheral blood as the stem cell source, and ≥1 comorbidity.

### 3.2 Clinical outcomes

**FIGURE 1:**
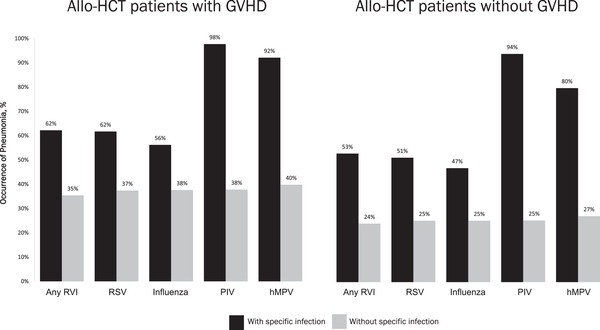
Occurrence of pneumonia in the first year after allo‐HCT for patients with and without a specific RVI stratified by GVHD status. All comparisons: *p *< .0001. allo‐HCT, allogeneic hematopoietic cell transplant; GVHD, graft‐versus‐host disease; hMPV, human metapneumovirus; PIV, parainfluenza virus; RSV, respiratory syncytial virus; RVI, respiratory viral infection

A significantly higher proportion of patients with any RVI experienced pneumonia within the first year following allo‐HCT compared to those with no RVI, irrespective of presence of GVHD (*p *< .0001; Figure 1). Similar significant findings were seen for each RVI evaluated (*p *< .0001 for all comparisons).

**FIGURE 2:**
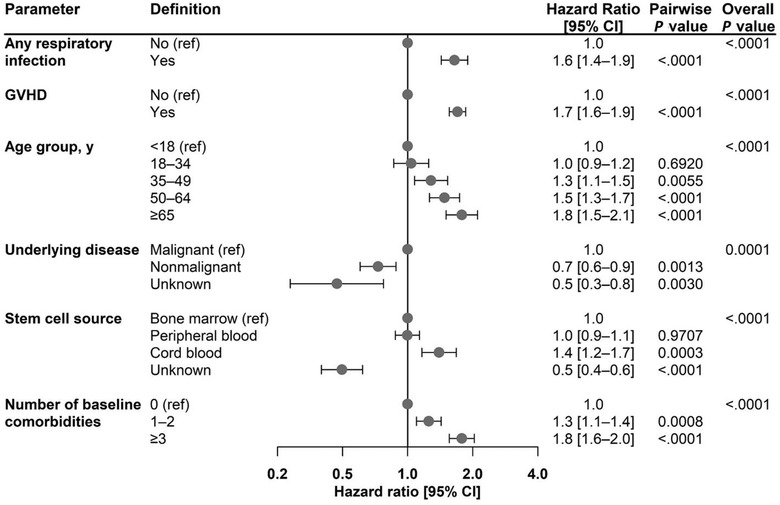
Cox proportional hazards model for time to all‐cause mortality for patients with and without RVIs. CI, confidence interval; GVHD, graft‐versus‐host disease; ref, reference; RVI, respiratory viral infection

In an adjusted analysis, patients with any RVI had a significantly higher risk of all‐cause mortality than those without an RVI (hazard ratio [HR], 1.6; 95% CI, 1.4–1.9; *p *< .0001; Figure 2). Presence of GVHD was associated with a significantly higher risk of all‐cause mortality (HR, 1.7; 95% CI, 1.6–1.9; *p *< .0001), as was increasing age ≥35 years (*p *< .01), cord blood stem cell source (*p *= .0003), and increasing number of comorbidities (*p *< .001).

### 3.3 Health resource utilization

**FIGURE 3:**
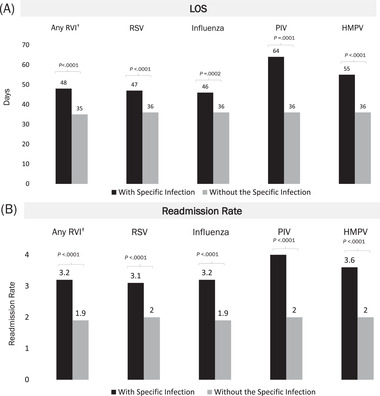
Health resource utilization within 1 year of undergoing allo‐HCT for patients with and without RVIs. Readmission rate defined as number of hospital readmissions after the index hospitalization/(number of days of follow‐up/365). For each comparison, patients without the specific infection could have a different double stranded deoxyribonucleic acid infection other than the one being examined. ^†^RSV, influenza, PIV, and hMPV. allo‐HCT, allogeneic hematopoietic cell transplant; hMPV, human metapneumovirus; LOS, length of stay; PIV, parainfluenza virus; RSV, respiratory syncytial virus; RVI, respiratory viral infection

Patients with any RVI had significantly higher health resource utilization (overall LOS, hospital readmission rate) compared to those with no RVI (*p *< .0001 for all comparisons; Figure 3). Significant findings were also seen for each RVI evaluated (all *p* ≤ .0002 for overall LOS and *p *< .0001 for readmission rate).

### 3.4 Health care reimbursement

**FIGURE 4:**
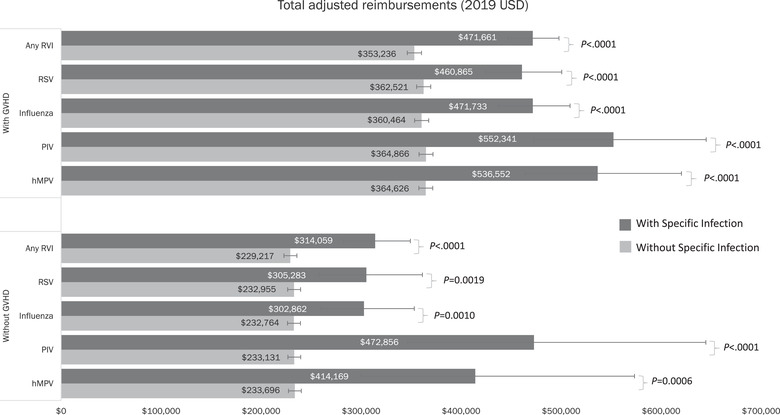
Total adjusted^†^ health care reimbursement in the first year after allo‐HCT for patients with and without an RVI stratified by GVHD status (2019 USD). ^†^Estimates adjusted for age, health insurance plan type, underlying disease, stem cell source, number of comorbidities, total reimbursements at baseline, GVHD during follow‐up, interaction between presence of RVIs and GVHD, and follow‐up time. allo‐HCT, allogeneic hematopoietic cell transplant; GVHD, graft‐versus‐host disease; hMPV, human metapneumovirus; PIV, parainfluenza virus; RSV, respiratory syncytial virus; RVI, respiratory viral infection

Patients with any RVI had significantly higher total health care reimbursement (unadjusted) within the first year after allo‐HCT compared to those with no RVI ($353 251 versus $220 856 USD, respectively; *p *< .0001; Table S2). Similar results were found for presence versus absence of each RVI analyzed (*p *< .0001 for all comparisons). Significant findings for total health care reimbursement (adjusted) were observed irrespective of presence of GVHD (*p *< .0001 for all comparisons of patients with GVHD; *p *< .01 for all comparisons of patients without GVHD; Figure 4).

## 4 DISCUSSION

This retrospective observational database study confirmed that patients with RVIs demonstrated significantly higher morbidity (i.e., pneumonia) and mortality, irrespective of GVHD presence. Health resource utilization, specifically overall hospital LOS and readmission rate, was also significantly higher in those with RVIs. Consistent with these findings, total health care reimbursement was significantly higher for patients undergoing allo‐HCT with an RVI compared to those without, with significantly higher total reimbursement found for all RVIs evaluated (RSV, influenza, PIV, and hMPV).

Our findings of increased morbidity and mortality, health resource utilization, and reimbursed costs in allo‐HCT recipients with RVIs are consistent with previous reports. Consistent with the literature, patients with PIV had the highest rate of pneumonia.^19^ Given this information, it is not surprising that we found that PIV was associated with the longest hospital LOS. Interestingly, prior studies have noted that influenza has a longer hospital LOS.^20^, ^21^ PIV could have had the longest LOS in our study owing to the availability of antiviral therapy for influenza but not PIV. In terms of total health care reimbursement, we found that hMPV had the highest reimbursement, followed by PIV, RSV, and influenza. Prior studies found a similar hierarchy of economic burden, which again is likely explained by severity of disease and availability of antiviral therapy for influenza.^20^

Because of the potentially serious consequences of RVI following allo‐HCT and the risk of progression from URTI to LRTI, early detection is extremely important.^1^, ^19^ However, even with early detection, a dearth of available antiviral agents and the limited efficacy and potential toxicity of the majority of these agents highlight the need for additional antiviral options, as well as other preventative and treatment strategies. In addition to improving patient outcomes, these strategies could potentially reduce resource utilization and health care cost, which is especially true of preventative therapies that could be applied to the highest risk population.

Limitations of the current study are related to the use of administrative claims data, including the potential that coding limitations could have affected the accuracy of clinical outcomes, identification of the severity of GVHD, and that some events may not have been entered into the database, including events that occur outside of the hospital. Claims database studies may often be limited by changes in patient health plans. This limitation may not be an issue for our study, as the claims database continued to track patients despite changing payers. We also acknowledge that our study only includes 13 363 of the >48 000 allo‐HCTs performed in the United States from 2012 through 2017, and some of this difference could be attributed to these limitations of the database. An additional limitation of an open‐source claims database is that the total reimbursed amount must be calculated for all submitted charges using a reimbursement‐to‐charge ratio obtained from the available submitted and reimbursed claims information. Exclusion of patients who underwent procedures in an outpatient setting may also have impacted our findings.

In conclusion, our study found that allo‐HCT patients with an RVI had worse clinical outcomes and a significantly higher burden on the health care system in terms of health resource utilization and total reimbursement. The adjusted analyses showed that mortality and total reimbursement were higher for allo‐HCT patients with an RVI, regardless of the presence of GVHD. Antivirals and other strategies may improve patient outcomes and reduce health resource utilization and costs after allo‐HCT.

## CONFLICT OF INTEREST

Michael G. Ison reports research support, paid to Northwestern University, from GlaxoSmithKline and Pulmocide; is a paid consultant for Adagio, ADMA Biologics, AlloVir, Cidara, Genentech, Roche, Janssen, Shionogi, Takeda, and Viracor Eurofins; is a paid member of DSMBs for AlloVir, CSL Behring, Janssen, Merck, Sequiris, Talaris, and Takeda; and receives royalties from UpToDate. Seung Hyun Moon is an employee of AlloVir and reports equity in AlloVir. Zhiji Zhang and Aastha Chandak are employed by an organization that received funding to conduct this study. Other authors have no conflict of interest to disclose.

## Supporting information



Supporting Information

Graphical Abstract

## References

[1] Pochon C , Voigt S . Respiratory virus infections in hematopoietic cell transplant recipients. Front Microbiol. 2018;9:3294. 10.3389/fmicb.2018.03294 30687278 PMC6333648

[2] Fontana L , Strasfeld L . Respiratory virus infections of the stem cell transplant recipient and the hematologic malignancy patient. Infect Dis Clin North Am. 2019;33(2):523‐544. 10.1016/j.idc.2019.02.004 30940462 PMC7126949

[3] Chemaly RF , Ghosh S , Bodey GP , et al. Respiratory viral infections in adults with hematologic malignancies and human stem cell transplantation recipients: a retrospective study at a major cancer center. Medicine (Baltimore). 2006;85(5):278‐287. 10.1097/01.md.0000232560.22098.4e 16974212

[4] Chemaly RF , Hanmod SS , Rathod DB , et al. The characteristics and outcomes of parainfluenza virus infections in 200 patients with leukemia or recipients of hematopoietic stem cell transplantation. Blood. 2012;119(12):2738‐2745. 10.1182/blood-2011-08-371112 22246027

[5] Chemaly RF , Rathod DB , Couch R . Respiratory viruses. In: Safdar A , ed. Principles and Practice of Cancer Infectious Diseases. Humana Press; 2011:371.

[6] D'Angelo CR , Kocherginsky M , Pisano J , et al. Incidence and predictors of respiratory viral infections by multiplex PCR in allogeneic hematopoietic cell transplant recipients 50 years and older including geriatric assessment. Leuk Lymphoma. 2016;57(8):1807‐1813. 10.3109/10428194.2015.1113279 26699199

[7] Paulsen GC , Danziger‐Isakov L . Respiratory viral infections in solid organ and hematopoietic stem cell transplantation. Clin Chest Med. 2017;38(4):707‐726. 10.1016/j.ccm.2017.07.012 29128020 PMC7130790

[8] Renaud C , Campbell AP . Changing epidemiology of respiratory viral infections in hematopoietic cell transplant recipients and solid organ transplant recipients. Curr Opin Infect Dis. 2011;24(4):333‐343. 10.1097/QCO.0b013e3283480440 21666460 PMC3210111

[9] Shah DP , Ghantoji SS , Mulanovich VE , Ariza‐Heredia EJ , Chemaly RF . Management of respiratory viral infections in hematopoietic cell transplant recipients. Am J Blood Res. 2012;2(4):203‐218. 23226621 PMC3512176

[10] Whimbey E , Englund JA , Couch RB . Community respiratory virus infections in immunocompromised patients with cancer. Am J Med. 1997;102(3A):10‐18. 10.1016/s0002-9343(97)80004-6 10868137 PMC7172994

[11] Ljungman P , Ward KN , Crooks BN , et al. Respiratory virus infections after stem cell transplantation: a prospective study from the infectious diseases working party of the European group for blood and marrow transplantation. Bone Marrow Transplant. 2001;28(5):479‐484. 10.1038/sj.bmt.1703139 11593321

[12] Pereira MR , Pouch SM , Scully B . Infections in allogeneic stem cell transplantation. In: Safdar A , ed. Principles and Practice of Transplant Infectious Diseases. Springer; 2019:209‐226.

[13] Mehta RS , Rezvani K . Immune reconstitution post allogeneic transplant and the impact of immune recovery on the risk of infection. Virulence. 2016;7(8):901‐916. 10.1080/21505594.2016.1208866 27385018 PMC5160395

[14] Kohlmeier JE , Woodland DL . Immunity to respiratory viruses. Annu Rev Immunol. 2009;27:61‐82. 10.1146/annurev.immunol.021908.132625 18954284

[15] Schmidt ME , Varga SM . The CD8 T cell response to respiratory virus infections. Front Immunol. 2018;9:678. 10.3389/fimmu.2018.00678 29686673 PMC5900024

[16] Sim SA , Leung VKY , Ritchie D , Slavin MA , Sullivan SG , Teh BW . Viral respiratory tract infections in allogeneic hematopoietic stem cell transplantation recipients in the era of molecular testing. Biol Blood Marrow Transplant. 2018;24(7):1490‐1496. 10.1016/j.bbmt.2018.03.004 29530766 PMC7110577

[17] Chemaly RF , Shah DP , Boeckh MJ . Management of respiratory viral infections in hematopoietic cell transplant recipients and patients with hematologic malignancies. Clin Infect Dis. 2014;59(5):S344‐S351. 10.1093/cid/ciu623 25352629 PMC4303052

[18] Ison MG , Hayden FG , Hay AJ , et al. Influenza polymerase inhibitor resistance: assessment of the current state of the art ‐ a report of the isirv antiviral group. Antiviral Res. 2021;194:105158. 10.1016/j.antiviral.2021.105158 34363859 PMC9012257

[19] Ison MG , Hirsch HH . Community‐acquired respiratory viruses in transplant patients: diversity, impact, unmet clinical needs. Clin Microbiol Rev. 2019;32(4):e00042‐00019. 10.1128/CMR.00042-19 PMC7399564 31511250

[20] Ghantoji SS , Shah DP , Lairson DR , et al. The economic and clinical burden of respiratory viral infections in hematopoietic cell transplant (HCT) recipients: a cost comparison study across 19 major cancer centers in the US. Biol Blood Marrow Transplant. 2016; 22:S53.

[21] Ghantoji SS , Karanth S , El Haddad L , Park A , Lairson D , Chemaly RF . Clinical and economic burden of respiratory viral infections in hematopoietic stem cell transplant recipients: the MD Anderson experience. Open Forum Infect Dis. 2017;4(1):S319‐S319. 10.1093/ofid/ofx163.748

