# Supplementary material for: Economic and clinical burden associated with respiratory viral infections after allogeneic hematopoietic cell transplant in the United States
Source: Transpl Infect Dis. 2022 Jun 1;24(4):e13866. doi: 10.1111/tid.13866 (PMC9542538; doi:10.1111/tid.13866)
Supplement: Supplementary file 1 — Supporting Information [file TID-24-e13866-s001.docx]

**Supporting Information**

**Table S1. Demographic and Transplantation Characteristics**

**Table S2. Total Health Care Reimbursement (Unadjusted) Within 1 Year of Undergoing Allo-HCT for Patients With and Without an RVI (2019 USD)**

**Figure S1. Study population.**

**Table S1. Demographic and Transplantation Characteristics**

|  | RVI Within 1 Year of Allo-HCT | |
| --- | --- | --- |
|  | **With Infection**  **(n=1368)** | **Without Infection**  **(n=11,995)** |
| Age at index allo-HCT, y |  |  |
| Mean (SD) | 44.4 (21.3) | 46.7 (20.7) |
| Range | 0 – >75 | 0 – >75 |
| Sex, n (%) |  |  |
| Female | 587 (42.9) | 5137 (42.8) |
| Underlying disease, n (%) |  |  |
| Malignant | 1221 (89.3) | 10,621 (88.6) |
| Acute leukemia | 674 (49.3) | 6073 (50.6) |
| Chronic leukemia | 63 (4.6) | 473 (3.9) |
| Lymphomas | 194 (14.2) | 1580 (13.2) |
| Myelodysplastic and/or myeloproliferative diseases | 241 (17.6) | 1993 (16.6) |
| Multiple myeloma | 55 (4.0) | 483 (4.0) |
| Solid tumors | 2 (0.2) | 39 (0.3) |
| Other | 139 (10.2) | 1420 (11.8) |
| Nonmalignant | 115 (8.4) | 1095 (9.1) |
| Unknown | 32 (2.3) | 279 (2.3) |
| Stem cell source, n (%) |  |  |
| Bone marrow | 190 (13.9) | 1607 (13.4) |
| Peripheral blood | 981 (71.7) | 8423 (70.2) |
| Cord blood | 107 (7.8) | 688 (5.7) |
| Unknown | 90 (6.6) | 1277 (10.7) |
| Number of baseline comorbidities, n (%) |  |  |
| 0 | 158 (11.6) | 2152 (17.9) |
| 1–2 | 666 (48.7) | 5954 (49.6) |
| ≥3 | 544 (39.8) | 3889 (32.4) |

Abbreviations: allo-HCT, allogeneic hematopoietic cell transplantation; RVI, respiratory viral infection.

**Table S2.** **Total Health Care Reimbursement (Unadjusted) Within 1 Year of Undergoing Allo-HCT for Patients With and Without an RVI (2019 USD)**

| **Observed total Health Care Reimbursement** | **RVIs within 1 year of allo-HCT** | | | ***P* Value** |
| --- | --- | --- | --- | --- |
|  | **With Infection** | **Without Infection** | **Difference in Medians** |  |
| Any RVI^†^ | n=1368 | n=11,995 |  |  |
| Mean (SD) | 476,961 (418,346) | 342,860 (392,824) | 134,101 | <.0001 |
| Median (Q1; Q3) | 353,251 (178,189; 637,219) | 220,856 (95,303; 439,876) | 132,395 | <.0001 |
| RSV infection | n=578 | n=12,785 |  |  |
| Mean (SD) | 490,851 (434,052) | 350,695 (395,484) | 140,156 | <.0001 |
| Median (Q1; Q3) | 365,600 (194,047; 657,393) | 226,161 (99,197; 450,597) | 139,439 | <.0001 |
| Influenza infection | n=687 | n=687 |  |  |
| Mean (SD) | 468,372 (424,020) | 350,508 (395,014) | 117,864 | <.0001 |
| Median (Q1; Q3) | 328,762 (153,768; 628,193) | 226,799 (98,863; 449,737) | 101,963 | <.0001 |
| PIV infection | n=166 | n=13,197 |  |  |
| Mean (SD) | 549,093 (463,612) | 354,346 (396,741) | 194,747 | <.0001 |
| Median (Q1; Q3) | 414,671 (245,157; 709,668) | 229,630 (100,582; 454,503) | 185,041 | <.0001 |
| hMPV infection | n=181 | n=13,182 |  |  |
| Mean (SD) | 561,960 (385,655) | 353,878 (397,355) | 208,082 | <.0001 |
| Median (Q1; Q3) | 477,463 (257,397; 773,445) | 229,434 (100,462; 453,161) | 248,029 | <.0001 |

allo-HCT, allogeneic hematopoietic cell transplantation; hMPV, human metapneumovirus; PIV, parainfluenza virus; Q1, 1^st^ quartile; Q3, 3^rd^ quartile; RSV, respiratory syncytial virus; RVI, respiratory viral infection.

^†^Includes RSV, influenza, PIV, and hMPV.

**Figure S1. Study population.**


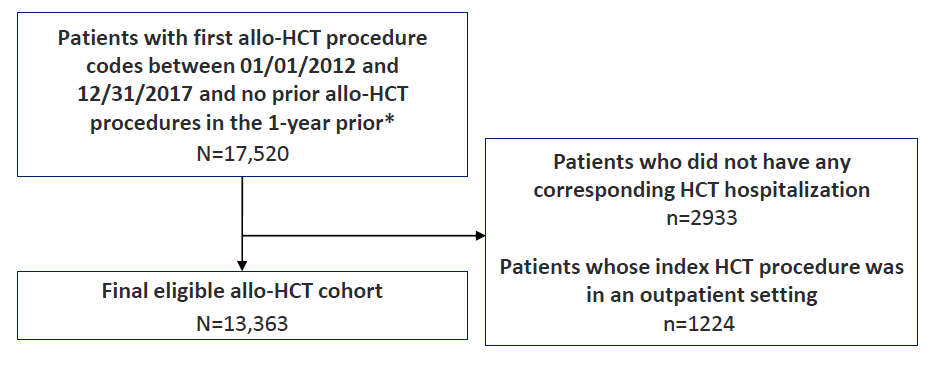


allo-HCT, allogeneic hematopoietic cell transplant.

*Note: Data were available from 01/01/2011 to 12/31/2018 but patients were identified between 01/01/2012 and 12/31/2017 to ensure that all patients have a 1-year baseline and 1-year follow-up period.
