# Supplementary material for: Economic and clinical burden associated with respiratory viral infections after allogeneic hematopoietic cell transplant in the United States
Source: Transpl Infect Dis. 2022 Jun 1;24(4):e13866. doi: 10.1111/tid.13866 (PMC9542538; doi:10.1111/tid.13866)
Supplement: Supplementary file 2 — Graphical Abstract [file TID-24-e13866-s002.pptx]

## Slide 1
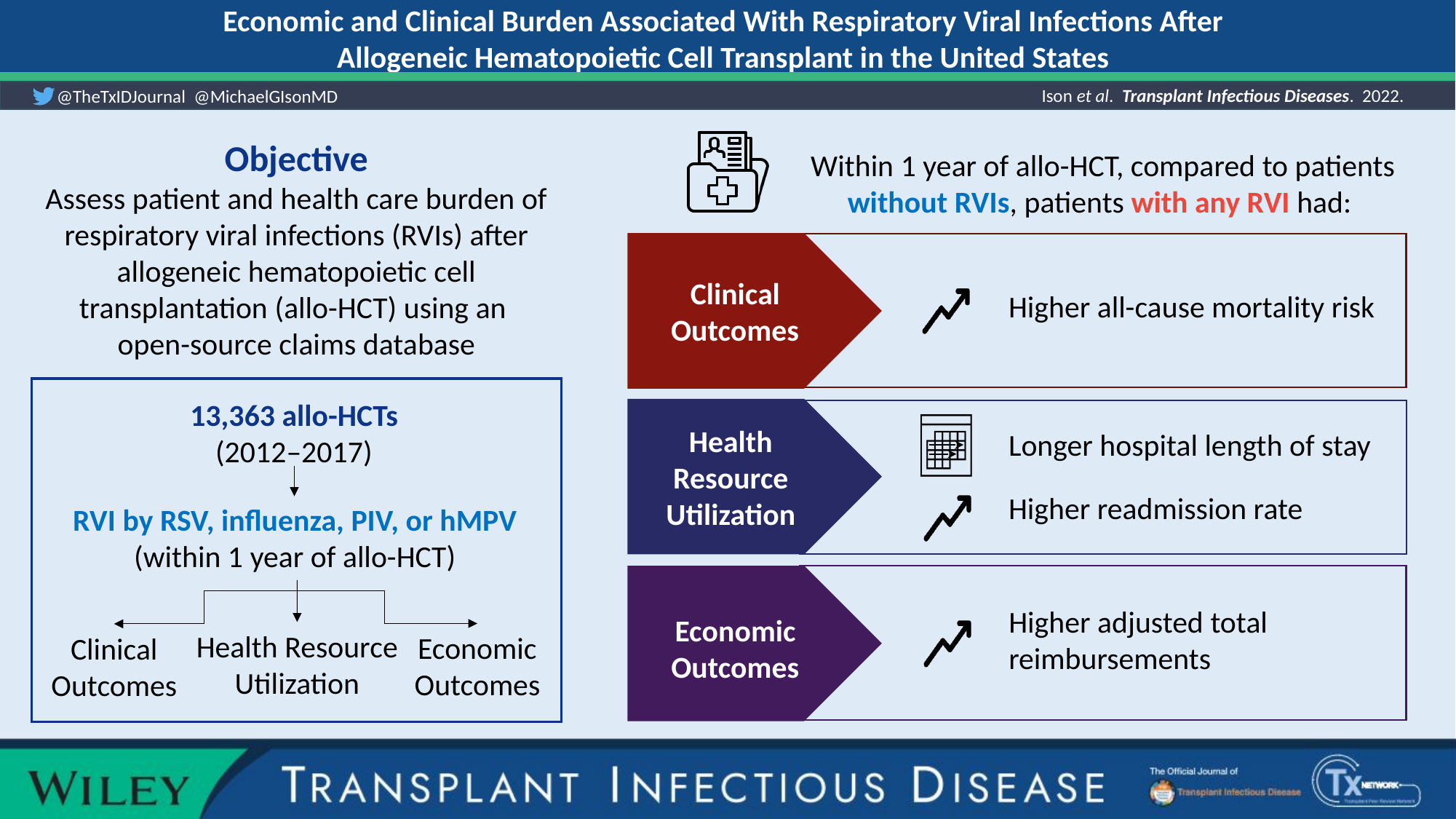

Economic and Clinical Burden Associated With Respiratory Viral Infections After
Allogeneic Hematopoietic Cell Transplant in the United States
Ison et al. Transplant Infectious Diseases. 2022.
 @TheTxIDJournal @MichaelGIsonMD
Objective
Assess patient and health care burden of respiratory viral infections (RVIs) after allogeneic hematopoietic cell transplantation (allo-HCT) using an
open-source claims database
Within 1 year of allo-HCT, compared to patients without RVIs, patients with any RVI had:
Clinical Outcomes
Higher all-cause mortality risk
Health Resource Utilization
Longer hospital length of stay
Higher readmission rate
13,363 allo-HCTs
(2012–2017)
Health Resource Utilization
Economic Outcomes
Clinical Outcomes
RVI by RSV, influenza, PIV, or hMPV
(within 1 year of allo-HCT)
Higher adjusted total reimbursements
Economic Outcomes
